# Simulation-assisted design of microfluidic sample traps for optimal trapping and culture of non-adherent single cells, tissues, and spheroids

**DOI:** 10.1038/s41598-017-00229-1

**Published:** 2017-03-21

**Authors:** Nassim Rousset, Frédéric Monet, Thomas Gervais

**Affiliations:** 1 Department of Engineering Physics, Polytechnique Montréal, PO Box 6079, station Centre-Ville, Montreal, H3C 3A7 QC Canada; 2 Institut de génie biomédical (IGB), Polytechnique Montréal, PO Box 6079, station Centre-Ville, Montreal, H3C3A7 QC Canada; 3 Institut du cancer de Montréal (ICM), Centre de recherche du Centre hospitalier de l’Université de Montréal (CRCHUM), 900, rue Saint-Denis, Montreal, H2X0A9 QC Canada

## Abstract

This work focuses on modelling design and operation of “microfluidic sample traps” (MSTs). MSTs regroup a widely used class of microdevices that incorporate wells, recesses or chambers adjacent to a channel to individually trap, culture and/or release submicroliter 3D tissue samples ranging from simple cell aggregates and spheroids, to *ex vivo* tissue samples and other submillimetre-scale tissue models. Numerous MST designs employing various trapping mechanisms have been proposed in the literature, spurring the development of 3D tissue models for drug discovery and personalized medicine. Yet, there lacks a general framework to optimize trapping stability, trapping time, shear stress, and sample metabolism. Herein, the effects of hydrodynamics and diffusion-reaction on tissue viability and device operation are investigated using analytical and finite element methods with systematic parametric sweeps over independent design variables chosen to correspond to the four design degrees of freedom. Combining different results, we show that, for a spherical tissue of diameter *d* < 500 μm, the simplest, closest to optimal trap shape is a cube of dimensions *w* equal to twice the tissue diameter: *w* = 2*d*. Furthermore, to sustain tissues without perfusion, available medium volume per trap needs to be 100× the tissue volume to ensure optimal metabolism for at least 24 hours.

## Introduction

**Figure 1:**
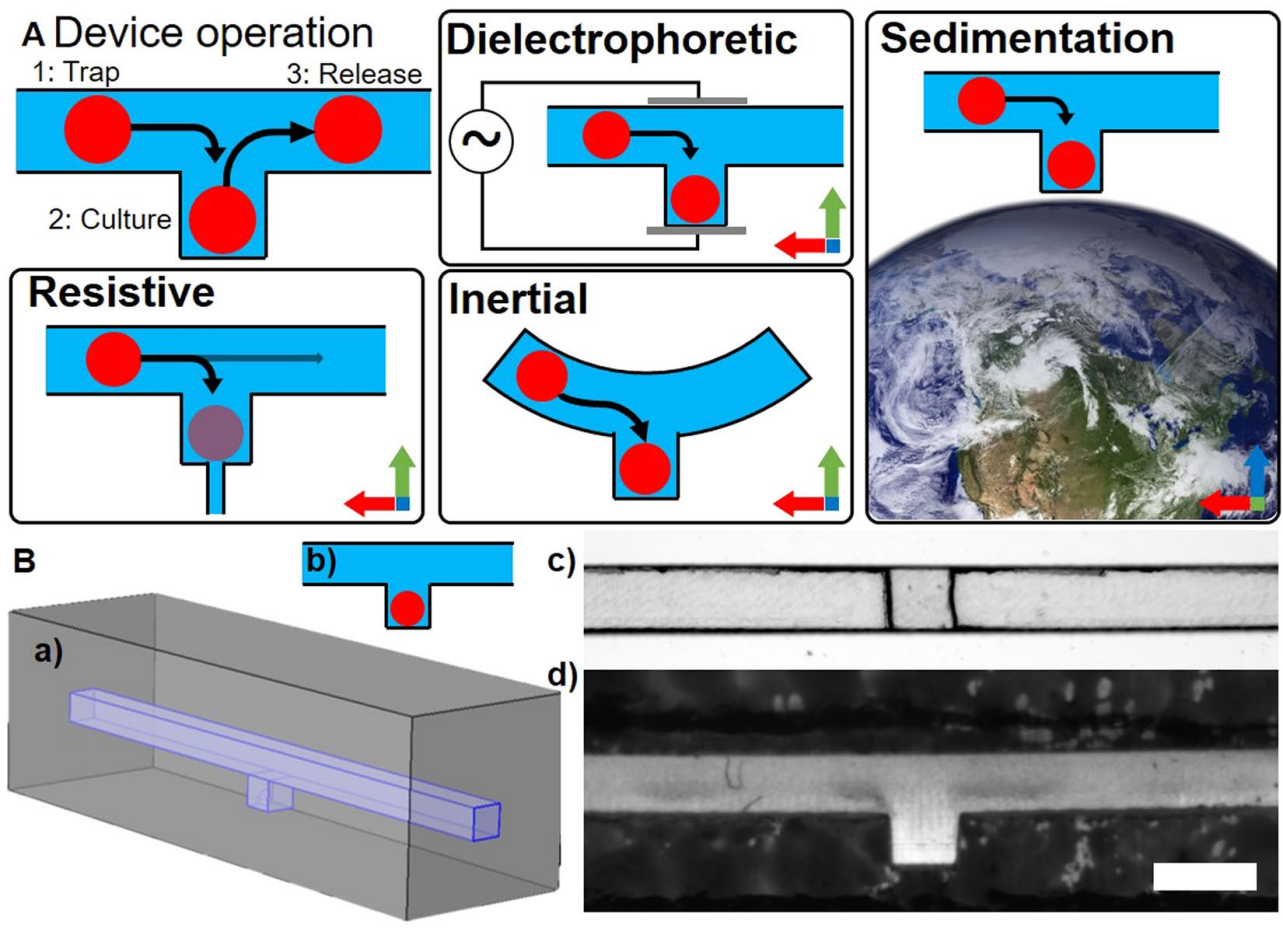
Basic MST designs: (**A**) MST operation with schematics of different trapping mechanisms: resistive, dielectrophoretic, inertial and sedimentation trapping; blue axis is parallel to gravity. The picture of the western hemisphere of earth on a transparent background was solely created by NASA and is in the public domain in the United States. (**B**) Simplified 3D (a) and 2D (b) MST design with a top (c) and side (d) view of a PDMS chip with these features; scale bar is 1000 μm.

Three-dimensional tissue models, ranging from spheroids to *ex vivo* samples of larger xenografts or even human tissues, are currently under extensive development to provide more biologically relevant preclinical models for personalized medicine and biopharmaceutical research^1–3^. The field of microfluidics has so far provided a large number of technical solutions to facilitate the trapping, culture and analysis of these tissues^2, 4–6^. An increasingly popular class of device to achieve this is the microfluidic sample trap (MST). As it is defined herein, MSTs are any device that allow to individually trap micrometre to millimetre-scale cell and tissue samples in a channel recess, hanging drop, or small chamber adjacent to a main channel where samples circulate in a carrier fluid (Fig. 1). They can be used to load samples or simply grow them within the trap from injected cell suspensions as in spheroid synthesis. The trapping mechanisms to load samples within the device vary depending on the design (Fig. 1A) and can combine many mechanisms studied by different authors, such as i) sedimentation trapping, where waiting for the tissue to settle into an extrusion from a channel traps it^7–10^; ii) resistive trapping, which exploits preferential flow to guide samples into traps^11–16^; iii) inertial trapping, which uses focusing flows or sharp turns to trap samples in channel recesses^17–19^; iv) dielectrophoretic trapping, where the difference in permittivity between the fluid and tissue is exploited to trap it in an electric field^20, 21^; v) and open-microfluidic channel networks, which create hanging droplets in which samples are trapped or synthesized^22, 23^.

In most designs, the trap itself allows tissues to be somewhat shielded from potentially damaging shear stress because the fluid velocity in it is only a fraction of what it is in the adjacent channel^8^. Although reflow of channel fluid in the trap is minimal, the small scale of the device allows the diffusion of nutrients and other species to occur quickly from the channel to the trap (and, conversely, diffusion of waste from the trap to the channel) relative to culture times. The relatively small design dimensions also allow to put a large number of traps (>100) within the same channel^9^. Additionally, if the device is made with a material porous to air (e.g. PDMS), it allows oxygen to be supplied throughout the experiment without requiring medium perfusion; while supplying other nutrients and purging waste require perfusion or periodic medium change^24, 25^. Finally, making the device with a transparent material (e.g. glass, PDMS) allows observation and analysis of samples in the device by different types of microscopy and wide field imaging^26^. All of these points show how MSTs are particularly effective at sustaining, treating and analysing large numbers of samples simultaneously.

Furthermore, even without perfusion, the viability of *in vitro* or *ex vivo* 3D tissue model samples has been shown not to be affected for sufficiently small tissues and with periodic medium change^10, 24^. The present study focuses on 3D tissue model sample culture in the absence of perfusion for three reasons: (1) studying non-perfused devices provides a worst-case evaluation of tissue viability; (2) integrating flow pumps to perfuse hundreds of samples simultaneously inside an incubator can be a barrier to adopting the technology by non-specialized users; (3) controlling small devices with large external pumps is counterintuitive to the miniaturization process. In the case of devices that are designed to not require perfusion, convection-based transport and metabolite diffusion are decoupled because they happen on different timescales. Convective effects occur briefly during loading, unloading and medium changes, and diffusive effects occur throughout the experiment.

Although designs and their trapping mechanisms vary, the simplest is a cubic extrusion from a channel or a well as Fig. 1B shows in three dimensions (a) and two dimensions (b). Figure 1B shows a cross-section of such a simple sedimentation trap made with PDMS^10^ viewed from the top (c) and the side (d). Figure 1A shows the basics of this device’s operation in its most fundamental form. In all cases, the trapping mechanism is caused by volume forces (*F*_*V*_) acting on samples, yielding an effective force towards the trap. Loading 3D tissue model samples into the device is done through the channel by applying a flow rate at the entrance or suctioning at the outlet and waiting some time to let the samples enter the trap. With a non-perfused design, as is the case in this analysis, it is necessary to change the medium periodically to replenish nutrients and evacuate sample excretions in the channel as it is cultured. All these aspects of device operation (trapping times, flow rates and medium replenishment) must be tuned to ensure that the device itself does not bias tissue viability.

This article aims to simplify MST design by studying different physical constraints (settling times, shear stress, lift forces, nutrient availability) that depend on device design and affect its operation (applied flow rates and medium replenishment frequency). To do so, analytical models are developed and compared to numerical models solved with a combination of the finite element method and broad parametric sweeps over all main design variables. The hydrodynamic models study non-adherent sample trapping and ejection dynamics for samples of similar sizes to the trap. The various diffusion-reaction models use experimental data on metabolite transport kinetics compiled from relevant cell-based studies. With these parametric sweeps, the results can be applied to many devices because the concept of a channel containing extrusions or wells that permit the trapping, culture, analysis, and release of single cells, spheroids and tissues has been applied in diverse scales. We demonstrate how and why these devices work and how to design them to achieve optimal operation for specific applications.

## Finite element methodology

The finite element method (FEM) is used in this work to model the device and simulate the effect of device design on device operation and tissue viability. The 3D geometry as shown in Fig. 1B was drawn, meshed, and different effects were solved numerically with COMSOL Multiphysics® v.5.2 (COMSOL AB, Stockholm, Sweden) run on a calculation server with dual Intel Xeon E5-2695V2 (2.4 GHz 12 core CPU) and 128 GB of RAM (Cyberlogic, Montreal, Canada). The software was chosen for its ability to easily do and analyse parametric sweeps with the built-in tools. COMSOL’s built-in probe functions, reactive force operators and the customizable parametric sweeps with stop conditions were key tools to produce the results.

### Model parameters and variables

There are three classes of design parameters in this study: parameters that affect device operation, parameters that do not affect device operation and tissue parameters.

**Figure 2:**
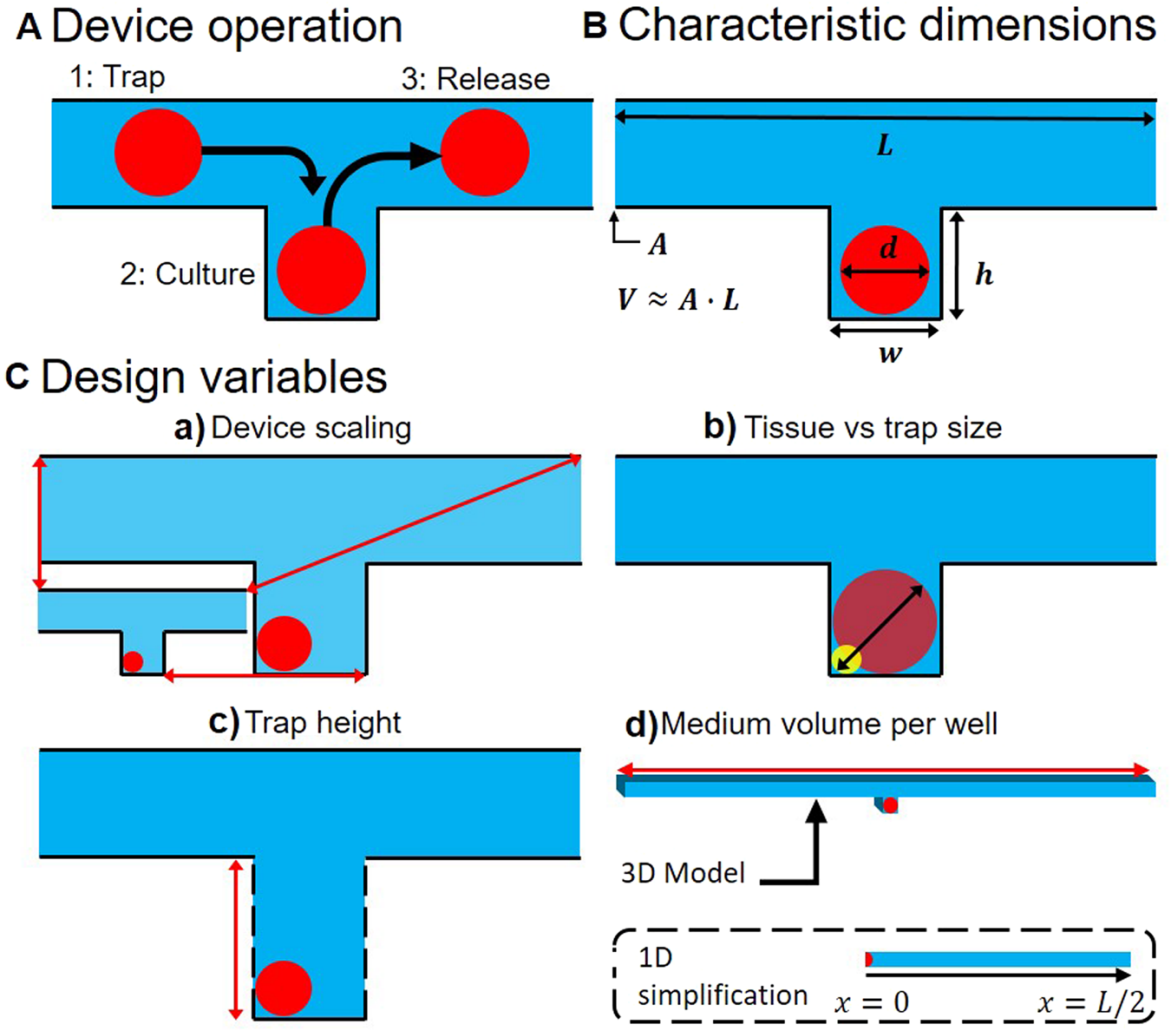
MST simulation methodology: (**A**) Sample traps operation schematic: (1) trapping of a tissue, spheroid or cell suspensions using any trapping mechanism (typically with volume forces), (2) culture, growth, treatment and *in*-*vitro* analysis of the tissues, and (3) release, ejection or collection of the samples from the traps for external analysis (e.g. flow cytometry). (**B**) Characteristic dimensions of the device with: *L* the inter-well separation, *A* the channel cross-section, *V* the available medium volume per well, *d* the tissue diameter (assuming a spherical tissue), *w* the well size (assuming a square well) and *h* the well depth. (**C**) Degrees of freedom for designing the device: (a) scaling the device up and down while keeping the tissue size constant compared to the well size, (b) scaling the tissue size while keeping all other dimensions constant, (c) scaling the trap height while keeping all other dimensions constant, and (d) scaling the available medium volume per well while keeping all other dimensions constant (inset shows the 1D simplification of the device for analysing diffusion of metabolites).

Device operation, meaning imposing a flow rate in the channel (to trap and release tissues or change channel medium) and refreshing metabolite concentration in the channel (to keep tissues alive), is affected by the following design parameters shown in Fig. 2B: well parameters as in well height (*h*) and well cross-section (*w* × *w*); and channel parameters as in channel length (distance between two traps) (*L*), channel cross-section (*A*) and channel volume (*V*). The available medium volume per trap *V*_*M*_ is approximately equal to the channel volume *V* if $L\gg w$.

The design parameter that does not affect device operation is PDMS thickness which is set at 3 mm, a value typically found in standard applications and not critical to derive most results.

The variability of tissue uptake parameters (tissue cellular density – inverse of cell size –, uptake rate and Michaelis-Menten constant) in literature is presented in Table 1 for two nutrients essential to metabolism: glucose and oxygen. The typical parameter values are taken from EMT6/Ro which are a fully characterized cell type for uptake and diffusion parameters. These parameters are pertinent but not essential for a diffusion analysis because they can be estimated in a worst-case approximation. For a fluid dynamics analysis, the only tissue parameter that is required is tissue diameter *d*, as shown in Fig. 2B, which is known by the type of tissue being trapped.

**Table 1:** Tissue uptake parameters found in literature for various cell types and the typical value used in the models.

Tissue uptake parameters		Minimum	Maximum	Typical
Tissue cellular density *ρ*_*cell*_ (cell/μL)		2.1 × 10^5^ ^47^	4.0 × 10^5^ ^48^	2.8 × 10^5^
Maximum cellular uptake rate *q*_*max*_ (mol/cells · s)	Glucose	2.7 × 10^−18^ ^49^	2.5 × 10^−16^ ^50^	3.9 × 10^−17^
	Oxygen	1.7 × 10^−18^ ^51^	7.0 × 10^−16^ ^52^	7.4 × 10^−17^
Michaelis-Menten constant *k*_*M*_ (mM)	Glucose	4 × 10^−2^ ^47^	6 × 10^−2^ ^49^	4 × 10^−2^
	Oxygen	4.6 × 10^−3^ ^50^	6.9 × 10^−3^ ^53^	4.63 × 10^−3^
Diffusion constant of O_2_ *D*_*x*_ (cm^2^/s)	Medium	2 × 10^−5^ ^46^	3.35 × 10^−5^ ^52^	2.44 × 10^−5^
	Tissue	9.5 × 10^−6^ ^29^	3.6 × 10^−5^ ^54^	1.85 × 10^−5^
Saturation concentration of O_2_ *c*_x-sat_ (mM)	Medium	—	—	0.21^10^
	Tissue	—	—	1.02^29, 55^

For diffusion parameters, oxygen diffuses through PDMS easier than through water^27, 28^ as the diffusion constant of PDMS (ranging from 3.25 × 10^−5^ cm^2^/s^27^ to 7.88 × 10^−5^ cm^2^/s^29^) is higher than that of water (ranging from 1.80 × 10^−5^ cm^2^/s at 20 °C to 2.78 × 10^−5^ cm^2^/s at 40 °C^30^). Thus, a simplified diffusion-reaction model of an oxygen consuming spherical tissue in an infinite sphere of water provides a worst-case analysis of oxygen supplied through the gas-permeable PDMS walls of the device. This simplified model approximates the *maximum viable tissue diameter* (*d*_*max*_) to prevent anoxia^10^:

1 $${d}_{\max }=2\sqrt{\frac{3}{{q}_{\max }{\rho }_{{cell}}}{(\frac{1}{2{D}_{T}{c}_{{T}-{sat}}}+\frac{1}{{D}_{M}{c}_{{M}-{sat}}})}^{-1}}\approx 500\,{\mu }{\rm{m}},$$

where *D* is the diffusion constant in the medium (*M*) or tissue (*T*), *c*_*sat*_ is the saturation constant, *q*_*max*_ is the maximum uptake rate and *ρ*_*cell*_ is the tissue cellular density in cells/μL. The value of *d*_*max*_ sets the maximum value for tissue size in the numerical simulations and parametric sweeps.

**Figure 3:**
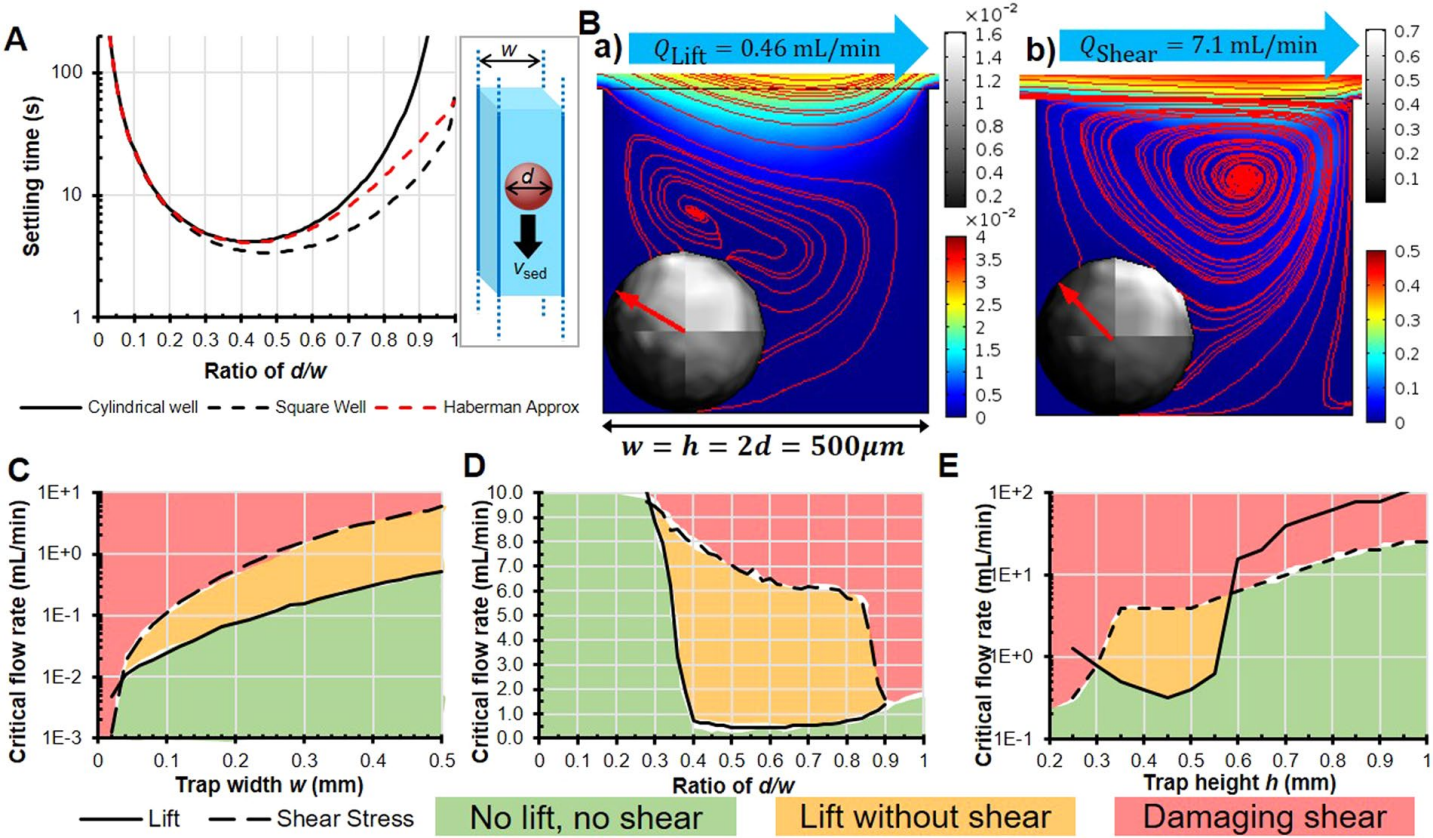
Hydrodynamic numerical results (**A**) Settling time as a function of the ratio of tissue diameter *d* over trap width *w* (as pictured in the inset) for two well cross-sections (cylindrical and square) compared to the analytical approximation by Haberman *et al*.^32^. Settling distance over a height of *w* (cubic well) for tissues 2% heavier than water. (**B**) Streamlines (red lines), flow velocity colour map (mm/s) in the well around the tissue, and shear stress greyscale map (Pa) on the tissue with the net resulting force of the flow on the tissue (red arrow) for a flow rate generating tissue lift *Q*_*lift*_ (a) and generating damaging shear stresses *Q*_*shear*_ (b). Phase diagram representation of critical flow rates for tissue ejection *Q*_*lift*_ and tissue shearing *Q*_*shear*_ for (**C**) varying trap width (or device scale) with a constant ratio *d*/*w* = 0.5 and *w* = *h* (result of ~5,800 simulations), (**D**) varying diameter over trap width ratios with *w* = *h* = 0.5 mm (result of ~4,300 simulations) and (**E**) varying trap height with a constant ratio *d*/*w* = 0.5 and *w* = 0.5 mm (result of ~1250 simulations).

**Figure 4:**
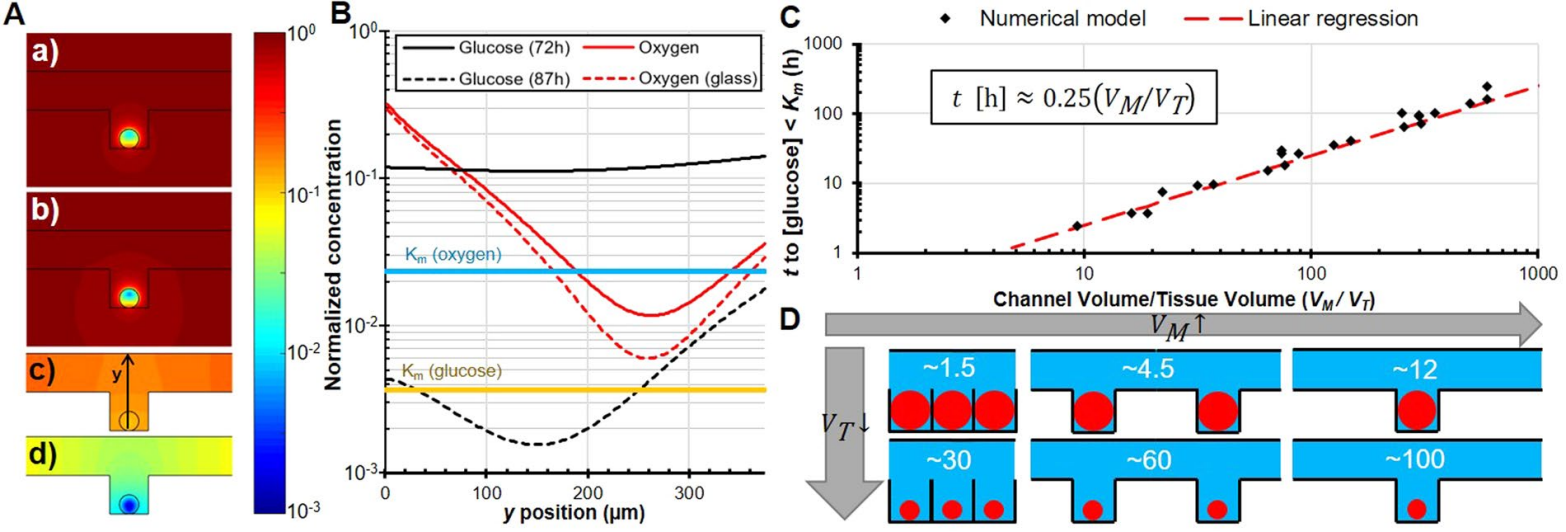
Spatial distribution of metabolites for *V*_*M*_/*V*_*T*_ ≈ 300, *d* = 375 μm and *w* = 750 μm (**A**) Colour mapping of the distribution of (a) oxygen in a full PDMS device, (b) oxygen in a PDMS device set on a glass slide, (c) glucose after 3 days, and (d) glucose after 87 hours; all normalized by the saturation concentration. (**B**) Distribution of various nutrients through the tissue and their associated Michaelis-Menten constants. (**C**) Time in hours before the minimum concentration of glucose in the tissue dips under the Michaelis-Menten constant, indicating a possibility in affecting cell metabolism as a function of the ratio of available volume per well on tissue volume (result of ~4,000 simulations). (**D**) Schematic of different available medium volume per well (*V*_*M*_) on tissue volume (*V*_*T*_) ratios.

To study the effect of design parameters that affect device operation, they must be modified independently with broad parametric sweeps in the numerical models that lead to as many degrees of freedom of the analysis as there are design parameters. These degrees of freedom must be minimized to a set of core “design variables” (DV) to reduce redundancies. Figure 2C shows the DVs circumscribed in this article. The primary DV is scaling the device down from its maximum size determined by *d*_*max*_ (Fig. 2C(a)), which adjusts the device for applications going from single-cell trapping to millimetre-scale 3D tissue model sample trapping. The secondary DV is varying tissue size within a trap of given dimensions (Fig. 2C(b)), which gives insight on sample behaviour that have a deviation from average dimensions since tissues are never all uniform in size. The tertiary DV is varying trap depth (Fig. 2C(c)), which describes sample behaviour based on the trap shape; a tissue in an elongated trap will behave differently from a tissue in a shallow trap. The final DV that is used solely for diffusion models is the inter-well separation (Fig. 2C(d)) which governs how densely multiple traps can be juxtaposed. The broad parametric sweeps performed on the four DVs lead to four series of over a thousand simulations each that have been graphically represented in Fig. 3C through Fig. 3E and Fig. 4C. A small sample of one such series for the secondary DV is provided (see Supplementary video).

### Tissue trapping

Tissue trapping encompasses the dynamics of tissues settling in a liquid-filled trap due to a volume force ***F***_*V*_; with buoyancy, this force is ${{\boldsymbol{F}}}_{V}=({\rho }_{T}-{\rho }_{M}){V}_{T}{{\boldsymbol{g}}}_{{eff}}$. With *ρ* the density of the tissue (T) and medium (M), *V* the volume and ***g***_*eff*_ the effective acceleration. In this study, gravity is assumed as the driving force ***F***_*V*_ but could be replaced by any of the volume forces shown in Fig. 1A.

Sedimentation of a solid spherical tissue of diameter *d* in a reservoir with dimensions comparable to its diameter is more complex than that of a sphere falling in an infinite medium. The drag force on a settling sphere increases with the ratio of the sphere cross-section on the reservoir cross-section in the direction of sedimentation^31^. To model this effect, the reference frame is changed to that of the settling sphere^32^. A sphere settling in a long prism (Fig. 3A inset) thus becomes an immobile sphere in a moving long prism with a moving wall boundary condition. The reactive force on the tissue (i.e. drag force in this referential) is found numerically with the *reacf* operator built into COMSOL. In the case of computational fluid dynamics, this operator evaluates the net vector force of flow on boundary nodes. The reactive force is found by summing this operator over all boundary nodes of the tissue.

With a parametric sweep on the moving wall velocity, the numerically evaluated drag force must be equal to the driving force on the sphere at which point the moving wall velocity is considered equal to the tissue settling speed. This method results in finding the settling speed *v*_*set*_ comparable to equation (3) and shown in Fig. 3A.

### Effect of fluid dynamics on tissues

As the results in Fig. 3B show, when a flow rate is applied in the channel, the tissue is pinned upstream by fluid recirculation within the trap. Thus, fluid dynamics effects are modelled with the spherical tissue upstream in the trap. This phenomenon has been studied for smaller particles in similar geometries^8^. Similar experimental observations were made using Polyethylene Microspheres in sedimentation traps such as those of Astolfi *et al*.^10^ (see Supplementary video). Additionally, since the tissue is pinned upstream, as the tissue lifts it is increasingly driven by vertical flow, thus increasing lift. This means that once the tissue takes off, it is very quickly ejected from the trap. These results are summarized in Fig. 3B through Fig. 3E.

For the numerical models, except when specified otherwise, walls are set with no slip (Dirichlet) boundary conditions on fluid velocity. The device is operated with a constant flow (*Q*) at the inlet and no stress (*p* = 0 Pa) at the outlet.

#### Critical flow rates to generate potentially damaging shear stress (*Q*_*shear*_)

The maximum value of the numerically solved shear rate over the surface of the tissue, multiplied by the fluid’s dynamic viscosity, gives the maximum shear stress on the tissue. To find the critical maximum flow (*Q*_*shear*_), the inlet flow rate (*Q*) is swept up until the maximum value of the shear stress (*τ*) exceeds physiological levels of 1 Pa^33–35^. Above this conservative value, cells are considered damaged as shear stress affects cellular phenotype^36^ and may induce apoptosis^37^, inhibit proliferation^38^ or remove adherent cells^34^. This method results in numerically finding the critical maximum flow rate that induces damaging shear (*Q*_*shear*_).

#### Critical flow rates to lift tissues (*Q*_*lift*_)

With large enough channels or high enough flow rates, Reynolds numbers can climb to reach transitional flow regimes which may generate lift forces that eject tissues. If the critical flow rates to reach those regimes are of the same order as loading flow rates, trapping stability is affected negatively. In other circumstances, this lift effect can be desirable and exploited to intentionally eject tissues from their traps. Numerically evaluating the net vertical force of the fluid onto the tissue resting at the bottom of the trap gives the lift force experienced by the tissue. Similar to finding the drag force for settling tissues, using the reaction force operator built into COMSOL gives the net vector force on the tissue. The vertical component gives the lift force, the longitudinal component gives the pinning force and due to symmetry the transversal component is null. If the lift force exceeds the net force pulling the tissue down (gravitational pull and buoyancy), then the associated flow rate is marked as being *Q*_*lift*_. This method results in finding the minimum critical lift flow rate (*Q*_*lift*_) because it assumes tissues do not adhere to the surface. Partially adherent tissues require higher lift flow rates.

### Diffusion of metabolites to the tissue

As there is no perfusion, the tissue can settle randomly at the bottom of the trap. Thus, as it is the worst-case scenario for metabolite diffusion, numerical diffusion simulations are done with the sample in the middle (${D}_{{\rm{O2}}}$ in PDMS is greater than in water) and at the bottom (furthest from the channel) of the trap.

To model diffusive effects, except when specified, all walls are set with no-flux (Neumann) boundary conditions. All interface boundaries (Air/PDMS, PDMS/Medium and Medium/Tissue) are set with a continuity condition on metabolite concentration normalized by the saturation concentration in the material, and on diffusive flow through these interfaces. Boundaries that allow for medium to flow in and out (inlets and outlets) are set with a periodic boundary condition to account for the presence of other traps. The initial concentrations of metabolites are set to the experimental values (11 mM)^10^. Finally, the tissue consumes metabolite with a Michaelis-Menten kinetics^39^,

2 $$q(c({\boldsymbol{x}},t))={q}_{\max }\frac{c({\boldsymbol{x}},t)}{c({\boldsymbol{x}},t)+{k}_{M}}$$

where the values of the Michaelis-Menten constant (*k*_*M*_) and the maximum uptake rate (*q*_*max*_) are shown in Table 1.

#### Diffusion of continuously replenished metabolites

Continuously replenished metabolites (i.e. oxygen) are provisioned through all PDMS surfaces exposed to air. Setting a constant air concentration boundary condition on all these surfaces models the presence of continuously replenished metabolites. Setting a no-flux boundary condition on the surface right under the trap models the case where the device is bonded onto a glass slide. Although the maximum tissue diameter may not be reached within certain device geometries, the numerical value of the minimum oxygen concentration within the tissue gives an idea on the effect of device design on diffusion of continuously provisioned metabolites (Fig. 4A,B).

#### Diffusion of finite metabolites

Finite metabolites, those that can be depleted in a medium (i.e. glucose), are strictly provisioned through the channel and require a medium change to be replenished. Running a time-dependent solver on a tissue consuming only the initially available nutrients in the medium models the diffusion of finite metabolites. The time-dependent solver is stopped when the minimum concentration of nutrients in the tissue reaches the Michaelis-Menten constant (*k*_*M*_), the threshold commonly accepted under which concentration limits uptake kinetics^39^. At that concentration, the uptake rate of cells is reduced to half of that of cells with an abundance of nutrient (*q*_*max*_ with a zero order kinetic). The corresponding time is then plotted as a function of available medium volume per trap (*V*_*M*_) per tissue volume (*V*_*T*_) in Fig. 4C.

## Numerical Models and Results

In this section, the Finite Element Method is used to solve the numerical simulations defined previously to describe different properties of device operation and design. The results for tissue trapping, fluid mechanics and nutrient diffusion are presented and compared to expected analytical results when available.

### Sedimentation trapping

The case of a sphere settling in an infinitely long cylindrical reservoir of diameter *w* was solved analytically and approximated numerically with the following equation^32^ and is shown in Fig. 3A as a function of *d*/*w*.

3 $$\begin{matrix}{v}_{{set}}(d/w) & \phantom{\rule{-1em}{0ex}}\approx \frac{1-0.76{(d/w)}^{5}}{1-2.1(d/w)+2.1{(d/w)}^{3}-1.7{(d/w)}^{5}+0.73{(d/w)}^{6}}\cdot \frac{1}{18}\frac{{\rho }_{T}-{\rho }_{M}}{\eta }{g}_{{eff}}{d}^{2}\end{matrix}$$

The settling time of tissues in gravitational traps is derived by dividing the length over which a tissue settles (*h*) by the settling speed of equation (3) and constitutes an upper bound on the experimental sedimentation time in a cubic trap. Bottom wall effects^40^ are neglected. The numerical results were derived for a settling length and trap size of *w* = *h* = 500 μm. As settling slowdown due to sample confinement is only a function of *d*/*w*, the shape of the curve is not affected by the trap size. It will, however, increase or decrease settling times inversely proportionally to the change (*t* ∝ *w* ^−1^). Decreasing the trap size by a factor of 10 will increase settling time by a factor of 10.

Figure 3A gives the complete information on settling times in gravitational traps. As expected, very small samples are exceedingly difficult to sediment due to their low weight and very large samples are exceedingly difficult to sediment due to increasing drag forces. This result shows that a square trap of a dimension between 1.4 and 3.3 times the tissue diameter (0.3 < *d*/*w* < 0.7) will minimize settling times. With this result, all further numerical simulations that do not require to vary tissue dimension are optimized with an average tissue to trap size ratio of *d*/*w* = 0.5.

### Viable design and operating windows

Figure 3C through Fig. 3E show the viable design and operating windows graphically. First, operating regions where tissues are subjected to damaging shear are highlighted in red. Second, optimal operating regions where tissues are removed from the device before being damaged are highlighted in orange. Third, operating regions where tissues remain in the device and aren’t damaged by shear are highlighted in green. A proper device usage ensures that shear stresses never exceed critical values in the trap. Therefore, assuming a non-adherent surface, an optimal design guarantees the tissue is ejected from the trap before these shear stresses are reached.

#### Minimum trap size

MST devices should be designed so that critical shear stresses are never reached within operating conditions. In effect, this means that the critical flow required to shear and damage tissues (*Q*_*shear*_) must be higher than the critical flow required to eject tissues from the device (*Q*_*lift*_). However, as the device is scaled down to smaller dimensions, *Q*_*shear*_ ∝ *w* ^3^ drops faster than ${Q}_{{lift}}\propto \sqrt{d}{w}^{2}$, as per equation (4). This means that there is a minimum trap width *w*_*min*_ under which tissues are potentially damaged by flow shear stress before being ejected from the device. Assuming a cubic trap with *h* = *w* and a tissue to trap size ratio of *d*/*w* = 0.5, equation (5) describes this.

4 $$[{Q}_{{shear}}=\frac{{\tau }_{\max }}{9.6\,\eta }{w}^{3}] > [{Q}_{{lift}}=\sqrt{d}{w}^{2}\sqrt{\frac{4}{3}g(\frac{{\rho }_{T}}{{\rho }_{M}}-1)}]$$

5 $$w > {w}_{\min }=61\frac{g{\eta }^{2}}{{\tau }_{\max }^{2}}(\frac{{\rho }_{T}}{{\rho }_{M}}-1)$$

*w*_*min*_ is considered the minimum trap size under which shear stress reaches 1 Pa before the tissue is lifted and is 12 μm with Table 1. With a tissue to trap size ratio of *d*/*w* = 0.5, *w*_*min*_ can be simulated numerically by scaling the entire device down from the maximum tissue diameter of *d*_*max*_ = 500 μm. Finding the crossing point of the critical flow rate for lift and the critical flow rate for shear stress as a function of the well width *w* gives that minimum trap size.

Figure 3C shows the numerical results of the simulations where the Haberman approximation is bounded by the square and cylindrical well results. These results consider a tissue of 1.02 g/cm^3^ density^10^ and are of the same order as the result in equation (5). A lighter or less compact tissue will result in a lower critical lift flow rate and minimum trap size. Conversely, a denser tissue will result in a higher minimum trap size. Although the results suggest to refrain from designing traps under the minimum trap size, if proper care is taken to limit flow in the device through external means, it is possible to make a device under the minimum trap size. The only drawback would be that tissues would not be extractable by applying flow and would require to dismantle the device or to design a separate extracting mechanism.

#### Tissue size variance

As all tissues are not exactly the same size, this section provides an analysis of tissue behaviour within the device for varying tissue dimensions. Similar to the previous section, ensuring that the device operates in a regime where damaging shear is impossible (*Q*_*shear*_ > *Q*_*lift*_) will lead to a more robust design. As the trapped tissue dimension changes within the device, the ease of shearing and ejecting it also changes.

Figure 3D highlights the design viability window in which the tissue is not subjected to damaging shear stresses but can still be ejected. Trapping tissues in too big (*w* > *d*/0.3) or too small (*w* < *d*/0.9) a device will lead to potentially damaging shear stresses before tissue ejection. This also shows that the *d*/*w* = 0.5 ratio proposed by settling times falls near the middle of this design viability window and it confirms that using it for numerical simulations does not lead to poor designs.

#### Trap aspect ratio

The shape and aspect ratio of the trap can also be tuned to fit specific applications. These simulations aim to study the effect of changing the well depth on the viability of tissues within a specific design. Figure 3E shows that increasing well height systematically impedes damaging shear stress. The reason for that can be seen from the colour mapping of Fig. 3B: the magnitude of fluid velocity in the trap decreases exponentially with depth. As the trap is elongated (increasing height), tissues become exponentially harder to shear and eject. However, as the trap is shortened (reducing height), tissues are more affected by flow in the channel and are easier to shear and eject. Figure 3E also has an inversion around *h* ≈ 1.2 *w* which shows that the optimal trap shape is a cube (or a trap with a height of similar scale to its cross-section).

### Optimal tissue metabolism

The main purpose of MST devices is to provide miniaturized culture platforms for assays involving 3D tissue models. Therefore, replenishing the device medium periodically is necessary to sustain and maintain high viability of 3D tissue model samples. Modelling how nutrients are consumed and diffuse in and around the tissue leads to a better understanding of these replenishment times and ensures that device design affects tissue viability the least.

The numerical simulations aim to estimate how long tissues can remain trapped without replenishing the medium and without affecting cell metabolism. For this, we focus on characterizing two nutrients essential to cell metabolism found in most culture media: oxygen and glucose. Since a Michaelis-Menten uptake kinetics is considered per equation (2), we set the nutrient threshold concentration to affect cell metabolism as the Michaelis-Menten constant *k*_*M*_. Although there are complex mathematical models of cell proliferation and death^41^, this threshold on cell metabolism is a subset of such models that leads to conservative replenishment times.

#### Metabolite spatial distribution

Figure 4A,B respectively show the distribution of nutrients through the device in 2D and through the tissue in 1D. This provides insight on diffusion mechanism and on the actual position in the tissue where the minimum nutrient concentration is. For the purpose of this section, a slightly larger device has been chosen, with *d* = 375 μm and *w* = 750 μm, to better see the effect of nutrient depletion and to demonstrate the worst-case scenario.

Figure 4A(a,b) shows that the bottom of the tissue has more oxygen due to oxygen diffusing easier through PDMS than through water. However, oxygen distribution in PDMS is nearly halved in the case of a device with a glass bottom. The non-porous glass slides upon which users sometimes set their devices have a dramatic effect on oxygen supply to biological samples. If a device relies on the porosity of the material used to supply oxygen, setting it on or bonding it to a glass slide is counter-intuitive.

The two-dimensional representation of nutrient distribution in Fig. 4A(c,d) shows that the bottom of the trap, or the side further from the channel, has less glucose. The shape of the trap does not affect much other than the position of the minimum nutrient concentration in the case of nutrients supplied exclusively through the channel medium as Fig. 4B shows.

#### Medium replenishment window

Under a certain channel length *L*_*max*_, channel shape does not significantly affect metabolite transport to the tissue. The nearly uniform glucose concentration of Fig. 4A(c) shows that diffusion occurs in similar or smaller timescales as nutrient uptake until glucose concentration reaches *k*_*M*_ as seen in Fig. 4A(d). Figure 4C shows that increasing tissue volume hastens glucose consumption, reducing the time required to affect cell metabolism *t*. Similarly, increasing the available medium per tissue increases the available glucose, increasing the time required to affect metabolism *t*. The linear regression of Fig. 4C follows the expected trend of equation (6) which was derived in previous work^10^ using the 1D simplification in Fig. 2B(c) inset for a regime that is not diffusion-limited.

6 $$t=a\frac{{c}_{0}}{{q}_{\max }{\rho }_{{cell}}}\frac{{V}_{M}}{{V}_{T}}+b$$

where *c*_0_ is the initial metabolite concentration, *V*_*M*_ is the available medium volume per tissue, *V*_*T*_ is the volume of consuming tissue, *q*_*max*_ *ρ*_*cell*_ is the maximum metabolite uptake in mol/m^3^s as shown in Table 1, and *a* and *b* are lumped proportionality constants that account for design geometry. For equation (6) to be valid, the longest dimension of the channel needs to be small enough (equation (7)) to stay out of a diffusion-limited regime. This is derived from a zero order Damköhler number *Da*_0_ = *t*_*diff*_/*t*_*up*_ using the total nutrient uptake time ${t}_{{up}}={c}_{0}{V}_{M}/{q}_{\max }{\rho }_{{cell}}{V}_{T}$ and the total nutrient diffusion time *t*_*diff*_ = *L* ^2^/8*D*_*M*_ where *Da*_0_ < 1 to remain out of a diffusion-limited regime. With a target *V*_*M*_/*V*_*T*_ = 100 (or *t* = 24 h), the threshold value *L*_*max*_ is approximated to 6.41 mm with the parameters of Table 1.

7 $$L < {L}_{\max }=\sqrt{8\frac{{c}_{0}{D}_{M}}{\,{q}_{\max }{\rho }_{{cell}}}\frac{{V}_{M}}{{V}_{T}}}$$

To use Fig. 4C, the desired maximum replenishment time and maximum tissue volume are required; in other words, the experimental frequency of medium replenishment and the largest trapped tissue samples. With the figure, medium replenishment time gives the target *V*_*M*_/*V*_*T*_ ratio and the largest expected *V*_*T*_ returns the required medium volume. Figure 4D shows different *V*_*M*_/*V*_*T*_ ratios for different trap densities and tissue sizes. In practice, two things must be considered: (1) as Fig. 4B shows, the region where metabolite concentration starts to affect metabolism is a small fraction of the tissue and (2) metabolite change takes some time to actually affect cell viability. This results in a practical wait time that can be significantly longer than the time in Fig. 4C without affecting tissue viability.

## Discussion

The previous numerical results provide a thorough example of how MSTs can be optimally designed and operated. This section compiles these results and summarizes the proposed design constraints of sample traps depending on the known sample characteristics. Table 2 summarizes the main design parameters as enumerated in the method section and shown in Fig. 2B.

**Table 2:** Device width *w* and height *h* compared to tissue diameter *d* to optimize device operation.

Device operation		Design dimension ranges	Optimal dimension
Minimum settling time when:		1.2*d* < *w* < 6.7*d*	*w* ≈ 2*d*
Sample ejection prior to reaching *τ* > 1 Pa	Minimum trap width	*w* > 30 μm	*Any*
	Trap width	1.1*d* < *w* < 3.3*d*	*w* ≈ 2*d*
	Trap aspect ratio	0.6*w* < *h* < 1.1*w*	*h* ≈ 0.9*w*
Maximum one sample per trap when:		*w* ≤ 1.4*d*	*w* ≈ 1.4*d*
Maximum tissue proliferation (24 h) when:		*V*_*M*_ ≥ 100 *V*_*T*_	*V*_*M*_ ≈ 100 *V*_*T*_

We conclude that the optimal dimensions of a trap are a base of 2*d* × 2*d* and a height slightly lower than 2*d*; in other words, a cube of side 2*d*. Although these dimensions are around 2*d* ≈ *w* ≈ *h*, this would potentially allow several tissues to be trapped within the same trap unless manually loaded. To ensure that only a single sample is allowed per cubic trap, the dimensions of the trap need to be slightly smaller (*w* ≤ 1.4*d*) than what is recommended in Table 2. Also, Fig. 3E shows that making a trap shallower or deeper than 0.9*w* reduces the operating window in which tissues are ejected before being sheared. This leads to a higher minimum trap size and more stringent operating windows for tissues of varying dimensions when $h\ne 0.9w$ compared to Fig. 3C,D respectively. Additionally, if the trap is too elongated, it will allow multiple samples to be trapped on top of each other regardless of trap dimensions, thus requiring user input to ensure single sample trapping. Opting for cubic or shallower traps solves this issue. Finally, since flow recirculation affects samples higher in the trap more strongly than samples at the bottom, it is possible to design traps that theoretically allow several samples but still trap single samples.

With a *d*/*w* ratio of 0.5 and *d* = 250 μm set at half the maximum tissue diameter, Table 3 emphasizes which aspects of device operation are affected by sample density. For the case of ejection flow rates, the Supplementary video concords with expected flow rate values (re-evaluated precisely for the experiment and available in the video caption).

**Table 3:** Comparing device design and operation for specific tissue densities.

Volumetric mass density (g/cm^3^)	1.01	1.1	2
Settling wait times (s)	≈10	≈1	≈0.1
Minimum device dimension (μm)	≈10	≈100	≈1000
Ejection flow rates (mL/min)	≈0.1	≈1	≈10
Shearing flow rates (mL/min)	≈7		

The effect of tissue density on device operation is function of the effective density, or tissue density minus medium density *ρ*_tissue_ − *ρ*_medium_. The only operating aspects that are affected by density require sample movement. Settling wait times and ejection flow rates are directly affected, whereas the minimum device dimension is affected due to that value being a crossing point between a *Q*_*lift*_ and a *Q*_*shear*_ curve. Finally, damaging shear stresses are not affected by tissue density. If tissues are too heavy (e.g. 2 g/cm^3^), it is possible to reach regimes where damaging shear stress occurs at lower flow rates than lift regardless of design. However, such high density cells or tissues do not exist: for example, single HeLa cells were found to have a density of ~1.043 g/cm^3^ ^42^.

Ultimately, accurately defining cell life and death or viability within a mathematical model is a challenging problem that has yet to be validated experimentally. Thus, this study focuses on establishing the time before cell metabolism or proliferation is expected to be affected. With Michaelis-Menten kinetics, this is associated with a reduction of cellular uptake of nutrients to half of the maximum value, or a nutrient concentration equal to the Michaelis-Menten constant. The time derived from the simulations and plotted in Fig. 4C helps establish a conservative replenishment time *t*_*rep*_. Following these recommendations ensures that cells throughout the tissue have an excess of nutrients at all times without perfusion by renewing cell medium at the interval dictated by *t*_*rep*_. In practice and since there is a certain time before cells react to nutrient change, it is possible to leave tissues longer than the suggested time in this work without significantly affecting cell viability.

To validate theoretical replenishment times *t*_*rep*_, the experimental methodology of several publications was investigated and compared with the model results. Table 4 compares the experimental *t*_*rep*_ to the modelled *t*_*rep*_ for various MST designs and tissue sizes. MST designs all fall within the few trapping mechanisms enumerated in Fig. 1. Tissue size varies from single cells to large tissue models. The selected publications all use human cancer cell lines of various sources (ovarian^9, 10, 23, 43^, prostate^10^, breast^11, 44^, liver^7, 18, 44, 45^, bone marrow^14^, colon^11, 22, 46^ and cervical^15^). The actual nutrient uptake parameters for each specific cancer cell line were not available in the literature, thus we used the typical values (Table 1) for EMT6/Ro as they provide a complete set of uptake and diffusion parameters. If the EMT6/Ro parameters overestimate the actual cell types’, the deduced replenishment times are more stringent than necessary.

**Table 4:** Comparing experimental and model replenishment times *t*_*rep*_ for several publications.

Trapping mechanisms	References	Cell Lines	Diameter of sample (μm)	$\tfrac{{{\boldsymbol{V}}}_{{\boldsymbol{M}}}}{{{\boldsymbol{V}}}_{{\boldsymbol{T}}}}$	*t*_*rep*_ (h)	
					Exp.	Model
Resistive	Frimat *et al*.^11^	SW580, HT29, MCF-7	17.9*	112	Perfusion	28
	Kukhtevich *et al*.^14^	K562	20*	127	Perfusion	31
	Occhetta *et al*.^15^	HeLa	16*	729	Perfusion	180
	Das *et al*.^43^^,§^	TOV112D	250^†^	138	24	34
Inertial	Ota *et al*.^18^	HepG2	450^†^	168	Perfusion	41
Sedimentation	Astolfi *et al*.^10^^,§^	22Rv1, PC3, TOV112D, OV90	380^‡^	208	48	52
	**Anada** ***et al***.^7^	**HepG2**	**600** ^†^	**52**	**24**	**13**
			310^†^	376	48	93
	Patra *et al*.^9^	TOV3041G, TOV112D, OV90, OV866(2)	130^†^	53	12	13
	Patra *et al*.^45^^,§^	HepG2	225^†^	92	24	23
Open microfluidics	Frey *et al*.^22^	HCT-116 eGFP	400^†^	470	Perfusion	116
	Marimuthu *et al*.^23^^,§^	OV90	500^†^	378	24	92
	Grimes *et al*.^46^	DLD1	585^†^	79	24	20
	Torisawa *et al*.^44^	MCF-7, HepG2	260^†^	165	48	41

*Single cells.

^†^ *Spheroids*.

^‡^ *Micro-dissected tissue*.

^§^ *Our group*.

Out of the 13 reviewed publications we found that 5 employed perfusion, thus automatically satisfying the modelled *t*_*rep*_ and ensuring optimal tissue metabolism; 4 had experimental *t*_*rep*_ under the modelled *t*_*rep*_, ensuring optimal tissue metabolism; 3 had experimental *t*_*rep*_ within 20% of the modelled *t*_*rep*_, causing glucose concentrations to briefly dip under *k*_*M*_ but not causing significant glucose deprivation; one had an experimental *t*_*rep*_ nearly twice the modelled *t*_*rep*_, affecting tissue growth rate. The latter (Anada *et al*.^7^) is the only publication with under-replenished tissues (highlighted in Table 4). As the tissue grows during the experiment reported in the publication, replenishment time is reduced from 48 to 24 hours after 6 days of incubation when spheroid size exceeds 310 μm. With the 24 hours replenishment time, spheroid growth rate slows down noticeable as tissue size passes the optimal *V*_*M*_/*V*_*T*_ ratio for 24 hours. With the aforementioned publication’s design and according to the model in this work, the maximum spheroid size for a 24 hour replenishment time to optimize cell metabolism through the whole spheroid is 487 μm. Exceeding that spheroid size leads to suboptimal cell metabolism in the spheroid core and, on a macroscopic scale, a slight reduction in spheroid growth rate.

The lack of under-replenished tissues in the literature is not surprising as tissue growth, and ultimately viability, should be affected in this case; biological results of under-replenished experimental methodology would then be less appealing for publication. Finally, in some cases where perfusion is used experimentally, Table 4 shows that periodical medium replenishment is possible while still keeping nutrient levels high; some require more stringent daily replenishment while some permit replenishment times of over 96 hours (4 days). The latter cases would benefit from using replenishment methods over bulky, contamination-prone perfusion systems.

## Conclusion

The previous analytical and numerical models have shown that optimizing tissue viability *in vitro* is possible and necessary to design effective MSTs. This article presents a complete and systematic analysis of all critical design variables of MSTs: trap size, tissue size, trap height and available medium per trap. Before culturing and analysing 3D tissue models in MSTs, ensuring that the device follows the specific design rules established in this research will confirm whether or not device design may affect the viability of samples.

When considering sedimentation, trapping stability and shear stress, the design rules in Table 2 prescribe that the best possible practical design is cubic traps at most twice the tissue diameter. When considering bonding to or using glass slides, the results show that they significantly impair oxygen transport and may lead larger tissues to anoxia. Finally, when considering nutrients that are only supplied through the medium, having a medium volume per trap that is at least 100 times the tissue volume with a maximum channel length of 6.4 mm ensures maximum metabolism (0^th^ order kinetics) for at least 24 hours. These *V*_*M*_/*V*_*T*_ ratio and maximum channel length can be tuned to the required channel design and desired replenishment times with equations (6) and (7). All of the results are derived for tissues with parameters listed in Table 1.

The prescribed design rules cover a wide range of devices made of porous materials (e.g. PDMS) with designs reducible to the simple MST geometry. These rules are consistent with published experimental studies utilizing MSTs. In some cases, perfusion is used where it would not be necessary^11, 14, 15^. In other cases, it is possible to have a slight reduction of viability when a suboptimal *V*_*M*_/*V*_*T*_ ratio is chosen^7, 10^. Ultimately, designs that aim to trap single cells or tissues should use nearly cubic traps at most twice the tissue size regardless of the material used for fabrication. In the case that the trap is larger, special mechanisms are put in place to ensure a single tissue per trap^15^.

The results presented in this article circumscribe a very specific but extensive set of experiments that will add to a better understanding of tissue viability *in vitro*. Validating these models requires precise knowledge of cell parameters: tissue density, cell density, Michaelis-Menten constants, uptake rate and tissue dimensions. These parameters can be evaluated experimentally for specific cell types with specialized equipment. The preceding design rules will enable designing MSTs as close as possible to optimal operating conditions, thus fabricating devices that fully exploit the potential of microfluidics to stably trap-release, and precisely control the *in vitro* tissue microenvironment.

## Electronic supplementary material

Supplementary info - video captions

Manipulation of polyethylene microspheres in a microfluidic sample trap device

Sample of the parametric sweep of flow rate magnitude in a device with varying “tissue” diameter
