# Supplementary material for: Simulation-assisted design of microfluidic sample traps for optimal trapping and culture of non-adherent single cells, tissues, and spheroids
Source: Sci Rep. 2017 Mar 21;7:245. doi: 10.1038/s41598-017-00229-1 (PMC5428016; doi:10.1038/s41598-017-00229-1)
Supplement: Supplementary file 1 — Supplementary info - video captions [file 41598_2017_229_MOESM1_ESM.pdf]

Simulation-assisted design of microfluidic sample traps for optimal trapping and culture of non-adherent single cells, tissues, and spheroids

Supplementary Information File

Nassim Rousset, Frédéric Monet, Thomas Gervais

Supplementary Video S1: Manipulation of polyethylene microspheres in a microfluidic sample trap device

Video of Polyethylene Microspheres (UVPMS-BG-1.025 355-425um) of 355 to 425 μm diameter and 1.026 g/cm^3 density (similar density and size as typical microtissues) being manipulated by flow rates within a typical PDMS microfluidic sample trap device. First, the upstream pinning of the spheres is shown (0:00 to 0:15). Then, a detailed view of the manual loading/ejection of spheres into/from traps is shown to emphasize that trapping occurs only when the flow is slow enough (0:15 to 1:00). Finally, the controlled ejection of spheres from traps is shown for flow rates ramping up from 1.0 to 1.4 mL/min (1:00 to 1:32). The channel and traps are 600×600 μm in cross-section and the trap is 500 μm in height. With this trap design, the models in the article predict a 0.78 to 1.14 mL/min ejection flow rate for the PE microspheres ($\rho = 1.026\ g/cm^3$, $d = 355$ to $425\ \mu m$) of the same order as the observed flow rates in the video.

Supplementary Video S2: Sample of the parametric sweep of flow rate magnitude in a device with varying "tissue" diameter

Video of a 2D cross-section of the 3D hydrodynamic solution within a microfluidic sample trap (MST) with tissue diameter from 300 to 450 μm in a MST of 500 μm trap width and height. This sample shows seven tissue diameters (300 to 450 μm by 25 μm intervals) with growing flowrates (1 to 20 $\mu L/s$ by 1 $\mu L/s$ intervals) for a total of 140 simulations out of the ~4,300 necessary to generate Figure 3D. Results shown are fluid streamlines and velocity magnitude colormap (in m/s) for every simulation result. The simulation solutions and the animation were produced with COMSOL Multiphysics® v.5.2 (COMSOL AB, Stockholm, Sweden). The lift force and shear stress are calculated with built-in COMSOL reactive force operators and probe functions which enable finding the critical lift Q_{lift} and damaging Q_{shear} flow rates plotted in figure 4.
